# Safety and Efficacy of Gliclazide as Treatment for Type 2 Diabetes: A Systematic Review and Meta-Analysis of Randomized Trials

**DOI:** 10.1371/journal.pone.0082880

**Published:** 2014-02-12

**Authors:** Gijs W. D. Landman, Geertruide H. de Bock, Kornelis J. J. van Hateren, Peter R. van Dijk, Klaas H. Groenier, Rijk O. B. Gans, Sebastiaan T. Houweling, Henk J. G. Bilo, Nanne Kleefstra

**Affiliations:** 1 Diabetes Centre Zwolle, Zwolle, The Netherlands; 2 Department of Epidemiology, University Medical Centre Groningen, Groningen, The Netherlands; 3 Department of General Practice, University Medical Centre Groningen, Groningen, The Netherlands; 4 Department Internal Medicine, University Medical Centre Groningen, Groningen, The Netherlands; 5 Department of Internal Medicine, Isala Clinics, Zwolle, The Netherlands; University of Catanzaro Magna Graecia, Italy

## Abstract

**Objective and Design:**

Gliclazide has been associated with a low risk of hypoglycemic episodes and beneficial long-term cardiovascular safety in observational cohorts. The aim of this study was to assess in a systematic review and meta-analysis of randomized controlled trials the safety and efficacy of gliclazide compared to other oral glucose-lowering agents (PROSPERO2013:CRD42013004156)

**Data Sources:**

Medline, EMBASE, Clinicaltrials.gov, Trialregister.nl, Clinicaltrialsregister.eu and the Cochrane database.

**Selection:**

Included were randomized studies of at least 12 weeks duration with the following outcomes: HbA1c change, incidence of severe hypoglycemia, weight change, cardiovascular events and/or mortality when comparing gliclazide with other oral blood glucose lowering drugs. Bias was assessed with the Cochrane risk of bias tool. The inverse variance random effects model was used.

**Results:**

Nineteen trials were included; 3,083 patients treated with gliclazide and 3,155 patients treated with other oral blood glucose lowering drugs. There was a considerable amount of heterogeneity between and bias in studies. Compared to other glucose lowering agents except metformin, gliclazide was slightly more effective (−0.13% (95%CI: −0.25, −0.02, I^2^ 55%)). One out of 2,387 gliclazide users experienced a severe hypoglycemic event, whilst also using insulin. There were 25 confirmed non-severe hypoglycemic events (2.2%) in 1,152 gliclazide users and 22 events (1.8%) in 1,163 patients in the comparator group (risk ratio 1.09 (95% CI: 0.20, 5.78, I^2^ 77%)). Few studies reported differences in weight and none were designed to evaluate cardiovascular outcomes.

**Conclusions:**

The methodological quality of randomized trials comparing gliclazide to other oral glucose lowering agents was poor and effect estimates on weight were limited by publication bias. The number of severe hypoglycemic episodes was extremely low, and gliclazide appears at least equally effective compared to other glucose lowering agents. None of the trials were designed for evaluating cardiovascular outcomes, which warrants attention in future randomized trials.

## Introduction

At present, metformin is the pharmacological cornerstone for patients with type 2 diabetes (T2DM) [1]. When metformin does not suffice or is contra-indicated, the next oral treatment options are; sulphonylureas (SUs), meglitinides, α-glucosidase inhibitor, thiazolidinediones (TZDs), dipeptidyl peptidase-4 (DPP-4) inhibitors, and sodium glucose transporter-2 receptor (SGLT-2) inhibitors. SUs are the preferred second treatment option in for example the current NICE guidelines, whereas no specific choices have been made in the American Diabetes Association and European Association for the Study of Diabetes (ADA-EASD) position statement [1], [2].

The new Dutch type 2 diabetes management guideline specifically advises gliclazide as the preferred second treatment option and not SUs as a group. Specifically advising gliclazide is – amongst others - based on evidence from observational studies showing cardiovascular benefits of gliclazide over other SUs [3]–[8]. Individual SUs express a different selectivity for pancreatic and myocardial SU receptors; gliclazide seems the most selective with respect to pancreatic receptor stimulation [7], [9]. Non-selective SUs like glibenclamide also block the myocardial SU receptor, and it is known that closure of these channels during myocardial ischemia worsens post-ischemic wall function by shortening the action potential [10]–[12]. Haemorheological and fibinolytic properties of gliclazide [13], a lower incidence of hypoglycemic events and less weight gain while using gliclazide were also proposed as explanations for a possible favorable cardiovascular safety profile [14]. Furthermore, no dose adjustments appears necessary in case of impaired renal function [15].

The role of gliclazide in the new Dutch guideline, the presumed differences between individual SUs, the possibility of a lower hypoglycemia risk, possible cardiovascular safety benefits together with the absence of a systematic review or meta-analysis examining gliclazide specifically, brought us to summarize the results from randomized studies comparing gliclazide with other oral glucose lowering agents.

## Methods

### Protocol

The eligibility criteria, outcomes, main and sensitivity analyses were pre-specified and published on PROSPERO (2013:CRD42013004156), see Attachment S1. The presentation of the methods and results is according to the PRISMA recommendations (available at www.prisma-statement.org).

### Eligibility criteria

A study was considered eligible if it was a randomized controlled trial that: treated non-pregnant adults (aged 18 or older) with type 2 diabetes; excluded patients during Ramadan; had a study duration of at least 12 weeks; reported change in glycated hemoglobin (HbA1c); compared gliclazide with other oral glucose lowering drugs. Trials comparing gliclazide with placebo, diet, insulin and rosiglitazone were excluded. The two preparations of gliclazide (gliclazide regular formulation and gliclazide MR) were grouped for all analyses.

### Data sources and searches

An electronic search without language restrictions was performed in Medline (using PubMed), EMBASE and the Cochrane Library (April 17 2013). *See Attachment S2. for our search strategy*. References list of selected articles were searched. Additional studies were searched for by hand searching the abstracts of the 2010, 2011 and 2012 annual meetings of the American Diabetes Association and the European Association for the Study of Diabetes. Completed but unpublished trials were searched (January 15, 2013) using websites of public registers of clinical trials (www.clinicaltrials.gov, www.trialregister.nl and www.clinicaltrialsregister.eu).

### Study selection

Publications retrieved from Medline, EMBASE, and the Cochrane Library, were imported in reference management software (www.endnote.com). After removing duplicate results, two authors (GL and KvH) independently performed the study selection. Differences between the reviewers were resolved by consensus with a third author (NK). One reviewer (GL) searched conference abstracts and trial registries.

### Data collection and data items

A data extraction form was designed and two reviewers (GL and PvD) independently abstracted data; discrepancies were resolved by consensus (see attachment). From each study, the following characteristics were extracted; author identification, year of publication, Clinical Trial number, sample size, type of intervention, duration of intervention, participants' baseline characteristics; age, sex, race, diabetes duration, previous treatment, HbA1c, body weight and pre-specified outcomes of efficacy and safety.

### Summary measures - Safety

The primary safety outcome was the number of patients experiencing at least one severe hypoglycemic event. A hypoglycemic event was considered severe when treatment by a third party was necessary.

Secondary safety outcomes included total number of confirmed non-severe hypoglycemic events, cardiovascular events and mortality. A non-severe confirmed event was defined as an event with symptoms and a glucose measurement with a plasma blood glucose <4.0 mmol/L. Cardiovascular events were defined as a combined endpoint of unstable angina, acute myocardial infarction, stroke and fatal cardiovascular events.

### Summary measures - Efficacy

The primary outcome was change in HbA1c from baseline to endpoint of the intervention, and its difference with the active comparator(s). The secondary efficacy outcome was the change in body weight from baseline to endpoint of the intervention. Time between baseline and endpoint measurements was considered as a covariate between studies.

### Subgroup analyses

Pre-specified subgroup analyses were planned regarding efficacy outcomes comparing gliclazide to other SUs/meglitinides, comparing gliclazide to other drugs except metformin, comparing gliclazide use as a second additive step in therapy and studies with at least 24 weeks follow-up. For the hypoglycemia analysis we planned a subgroup analysis in studies using a maximum gliclazide dose of 240 mg.

### Missing data and multiple reports

Data from intention to treat (ITT) (all participants randomized) or modified ITT (all randomized participants who received intervention and had at least one measurement after baseline) were used when these were available either in a published paper or on websites of pharmaceutical companies and trial registries.

In case of missing, incomplete or per protocol (PP) data regarding the primary and secondary outcomes, the corresponding authors were contacted (three attempts maximum). In case of not responding, the pharmaceutical companies, if applicable, were contacted and ITT or modified ITT data were requested.

In case of multiple reports or companion papers of the same study (published results of an extension period) outcome data from the original study were extracted, unless the first report was a planned interim analysis.

### Risk of bias assessment

The Cochrane Collaboration's risk of bias tool was used to assess risk of bias [16]. This tool considers the presence of bias caused by: random sequence generation, allocation concealment, blinding of participants and personnel, incomplete outcome data (because of high rate of discontinuation, type of analysis, or imputation of missing data), selective reporting and a category ‘other forms of bias’. Study registration became mandatory in 2004 [17], with September 13, 2005 as the last date for trials not registered at inception [18]. Studies without registration after this period were considered at high risk for selective reporting.

The risk of bias was regarded high in case of the presence of high bias in any domain, low if all key domains (all domains except random sequence generation and allocation concealment) were of low bias, and unclear in all other cases. When only the risk of bias in the 7th domain (other bias) was unclear it was regarded as low risk of bias [16], [19]. Two authors (GL and PvD) independently assessed the risk of bias, when necessary consensus was determined through help of a third author (GdB).

### Synthesis of results

Mean differences between the intervention group and all active comparator groups with standard deviations (SDs) were calculated for continuous outcomes with an inverse variance random effects model. If a specific study did not report standard deviations, this was calculated from the standard error or the 95% confidence interval (CIs). For dichotomous outcomes, risk ratios and 95% CIs were used, again using an inverse variance random effects model.

In the absence of a gliclazide group receiving the maximum approved dose of 240 mg, data regarding the group receiving the highest dose were analyzed.

Statistical heterogeneity (I^2^) values of 30–60% and between 60–90% represent moderate and considerable heterogeneity, respectively [16]. Potential causes of heterogeneity were explored by looking at outliers and by performing sensitivity analyses, excluding reports with high overall risk of bias. Sensitivity analyses were planned for every outcome based on its overall risk of bias.

All analyses were performed with RevMan 5.1 (Nordic Cochrane Centre).

## Results

**Figure 1:**
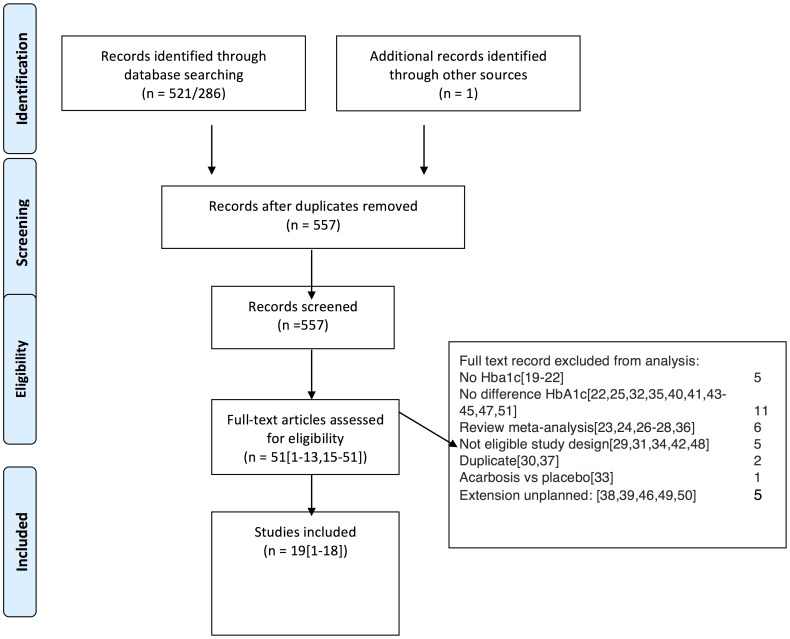
PRISMA flow diagram. Presentation of the procedure of literature searching and selection with numbers of articles at each stage.

In total, 19 studies were included (see figure 1). 18 studies were retrieved from searching in electronic databases [8], [14], [20]–[35] and one study, including Chinese patients, was selected through searching Clinicaltrials.gov [36]. No additional completed trials were retrieved from other sources. The ADVANCE study was not included because the primary goal of the study was to compare a strict control (with gliclazide) to conventional treatment (without gliclazide) and not gliclazide itself [37].

Although some authors or pharmaceutical companies responded [8], [27], [29], others either did not respond or did not give priority to our requests [26], [28], [30]– or stated that intention to treat analyses could not be performed for differences in weight [34], [35].

Table 1 summarizes the characteristics of the included studies. Participants' baseline characteristics show that the studies were heterogeneous in design. The durations of the intervention periods varied between 12 and 104 weeks. All but two studies used the regular gliclazide formulation [8], [29].

**Table 1:** Summarizes the characteristics of the included studies.

Primary study	Study arm (max dose)	Comparison arm (max dose)	Study duration (month)	HbA1c (%)	Age	Add on to:	N	Sex % female	Diabetes duration (years)	Weight (kg)
Harrower 1985	Gliclazide 320 mg	Glibenclamide 30 mg	52	12	60	No	20/19	n.a.	4	61
Jerums 1987	Gliclazide 240 mg	Glibenclamide 15 mg	104	9.6	60	No	9/8	35	8	n.a.
Collier 1989	Gliclazide 240 mg	Metformin 3 g	24	11.9	54	No	12/12	n.a.	0	n.a.
Noury 1991	Gliclazide 240 mg	Metformin 1.7 g	13	9.7	55	No	27/30	51	3	80
Tessier 1994	Gliclazide 320 mg	Glibenclamide 20 mg	26	8.6	72	No	11/11	18	5	n.a.
Tessier 1999	Gliclazide 320 mg	Metformin 1.7 g	24	7.5	59	No	18/18	31	5	82
Guvener 1999	Gliclazide 320 mg	Acarbose 600 mg	26	8.5	56	Insulin	18/20	79	11	n.a
Salman 2001	Gliclazide 320 mg	Acarbose 600 mg	24	8.8	54	No	30/27	42	4	na
NCT01022762 2010	Gliclazide 240 mg	Repaglinide 12 mg	16	7.2	62	Metformin	218/217	46	1	n.a.
Furlong 2003	Gliclazide 240 mg	Repaglinide 12 mg	13	9.3	59	Insulin	39/41	47	8	91
Lawrence 2004	Gliclazide 320 mg	Pioglitazone 45 mg Metformin 3 gr	24	7.7	61	Low dose oral glucose lowering agents 66%	20/20/20	35	n.a.	68
Schertnhamer 2004	Gliclazide 120 mg XR	Glimepiride 6 mg	27	8.3	61	Metformin/acarbosis	388/427	49	6	84
Charbonell 2004	Gliclazide 320 mg	Pioglitazone 45 mg	52	8.7	n.a.	No	1270 total	n.a.	n.a.	n.a.
Mettews 2005	Gliclazide 320 mg	Pioglitazone 45 mg	52	8.6	57	Metformine	313/317	50	6	92
Kardas 2005	Gliclazide MR 90	Glibenclamide 10 mg	16	7.2	62	Metformine in 28%	49/50	60	3	77
Pierriello 2006	Gliclazide 320 mg	Pioglitazone 45 mg	52	8.8	59	Diet or 1 oral drug	140/135	35	9	80
Ristic 2006	Gliclazide 240 mg	Nateglinide 180 mg	24	7.6	62	Metformin	133/129	48	7	n.a.
Foley 2009	Gliclazide 320 mg	Vildagliptine 100 mg	104	8.6	55	No	533/530	44	2	84
Filozof 2009	Gliclazide 320 mg	Vildagliptine 100 mg	52	8.5	59	Metformin	490/503	48	7	85

The primary end point between studies varied, the most common endpoint being change in HbA1c. Five studies had a non-inferiority design [29], [30], [34]–[36], four used a 95% confidence interval, with non-inferiority margins of 0.3% [34], 0.4% [35], [36] and 0.5% [29] and one used a 90%CI with a non-inferiority margin of 0.2% [30]. All 19 included studies contributed to the HbA1c analysis; 3,083 patients were randomized to gliclazide and 3,155 patients were randomized to another glucose lowering agent. From the studies that reported sponsoring, almost all were sponsored by a pharmaceutical company [27], [28], [30]–[36].

Gliclazide was compared to other SUs/meglitinides in eight studies [8], [14], [20], [21], [27], [29], [33], [36], to metformin in four studies [22], [23], [25], [28], to pioglitazone in four studies [28], [30]–[32], to DPP-4 inhibitors in two studies [34], [35] and to an α-glucosidase inhibitor in two studies [24], [26]. One study compared gliclazide to both metformin and pioglitazone [28].

In 12 studies [8], [22], [23], [26], [27], [29], [31]–[36] severe hypoglycemia, and in 7 studies [8], [14], [22], [23], [26], [34], [35] non-severe confirmed hypoglycemia could be evaluated.

Eight studies contributed to the weight analysis. Two studies with metformin [23], [25], one with glibenclamide [20], one with nateglinide [33], one with acarbose [26], one with pioglitazone [32] and 2 studies with repaglinide as a comparator [27], [36].

None of the trials was designed to assess cardiovascular safety and/or efficacy. In two larger studies, comparing gliclazide with glimepiride and pioglitazone respectively, specification of cardiovascular events was not possible. Therefore, they were not included in the meta-analysis [29], [30].

### Risk of bias

Overall risk of bias for the primary outcome was low in one study [29], unclear in one [24] and high in all others (see Attachment S3 and S4).

Few studies described differences in weight with SD's for the ITT or modified ITT populations [20], [23]–[27], [32], [33], [36]. The two studies with DPP-4 inhibitors were at high risk for incomplete outcomes (exclusion bias) [34], [35]. In both cases the PP analysis of weight were in favor of vildagliptin users [34], [35]. ITT data were not handed over. The study on nateglinide was subject to selective reporting by not reporting differences in weight and was also subject to a high risk of attrition bias [33]. In two studies, pioglitazone was accompanied with a higher weight increase compared to gliclazide users without making formal comparisons [30], [31]. Two studies with pioglitazone were at high risk for attrition bias [30], [31]. One pioglitazone study also had a high risk for detection bias [31]. Four large studies were published after 2005 [32]–[35]. These four studies were either not registered [32], [33] or not appropriately registered (before start of the recruitment phase) [34], [35] and were regarded at high risk for selective reporting (outcome bias).

### Severe hypoglycemia

In those studies, in which severe hypoglycemic events were systematically reported, there was one severe hypoglycemic event in 2,387 gliclazide users and one in the 2,430 patients in the comparator group [8], [22], [23], [26], [27], [29], [31]–[36]. The one severe hypoglycemic event in the gliclazide group occurred in a patient who also started NPH insulin in the prmonths preceding the incident [27]; the one severe hypoglycemic event in the comparator group occurred in a patient using glibenclamide.

### Confirmed non-severe hypoglycemia

Symptomatic confirmed hypoglycemia's could be evaluated in 7 studies [8], [14], [22], [23], [26], . There were 25 hypoglycemic events (2.2%) in 1,152 gliclazide users and 22 hypoglycemic events (1.8%) in 1,163 patients in the comparator group (risk ratio 1.09 (95% CI: 0.20, 5.78; NS) after 13 to 104 weeks follow-up. There was a high heterogeneity (I^2^ 77%) across studies.

Three out of 7 studies were responsible for all symptomatic confirmed hypoglycemic events [14], [34], [35]. Two studies comparing gliclazide with vildagliptine had a relative long follow-up period of 52 and 104 weeks and used a gliclazide dose of up to 320 mg, well above the official maximum of 240 mg [34], [35]. One small study comparing with glibenclamide showed 12 confirmed symptomatic hypoglycemic events in the glibenclamide group and 0 in the gliclazide group [14]. After excluding studies that used a dose above 240 mg, there were no symptomatic confirmed hypoglycemic events in 129 gliclazide users, these studies had a follow-up period between 13 and 26 weeks [14], [22], [23], [26].

One study, with low risk of bias, comparing gliclazide (XR 120 mg) with glimepiride showed an advantage regarding non-severe, confirmed symptomatic and confirmed asymptomatic hypoglycaemia episodes in favour of gliclazide. 15 patients experienced a hypoglycaemic episode in 403 gliclazide users compared to 39 patients in 439 glimepiride users (p<0.02) [29]. This study was excluded from the meta-analysis because asymptomatic episodes were also included in the study analysis (despite registered glucoses <3.0 mmol/L) [29].

### Glycemic efficacy

**Figure 2:**
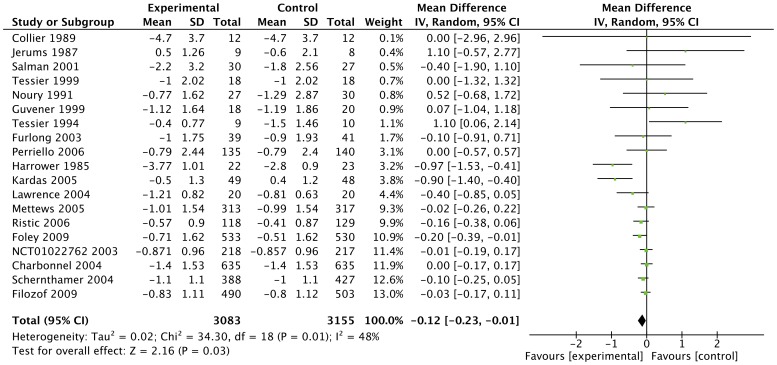
Forest plot of the main effect outcome. The main effect outcome HbA1c; gliclazide versus other glucose lowering agents. Metf = metformin, SU is sulphonylurea, Pio is pioglitazone.

Compared to all other interventions, gliclazide was more effective: −0.12% (95%CI: −0.23, −0.01) on the primary outcome measure; change in HbA1c from baseline (see Figure 2). There was moderate heterogeneity with I^2^ of 48%. The study by Kardas [8] and the study by Harrower [20] were mainly responsible for the heterogeneity. After excluding these studies, the effect estimates were −0.09 (95%CI: −0.18, −0.00).

After excluding 3 studies [22], [23], [25] that used metformin as comparator arm and the metformin comparator arm of the study by Lawrence [28], gliclazide was more effective: −0.13% (95%CI: −0.25, −0.02, I^2^ 55%).

In the 12 studies where the risk for severe hypoglycemia could be evaluated; gliclazide was more effective (−0.11% (95%CI: −0.2, −0.01, I^2^ 26%)). When comparing to other SUs, there was no significant difference in HbA1c: −0.21% (95% CI: −0.46, 0.05, I^2^ 74%). Inclusion of studies with a maximum dose set at 240 mg gliclazide [8], [21]–[23], [27], [29], [33], [36] resulted in a non-significant difference of −0.14% (95%CI −0.34, 0.05, I^2^ 50%), compared to all other interventions. After excluding studies that used gliclazide MR, the effect estimate was −0.09% (95%CI: −0.20, 0.02, I^2^ 35%). As second line treatment [24], [27], [29], [31], [33], [35], gliclazide was not significantly different to comparators: HbA1c −0.07% (95%CI: −0.16, 0.01, I^2^ 0%). After excluding studies with a duration of less than 24 weeks the effect estimate was −0.11% (95%CI: −0.21, −0.00, I^2^ 38%). Compared to metformin monotherapy, the effect estimate of gliclazide monotherapy was 0.26 (95%CI: −0.59, 1.11, I^2^ 0%).

### Weight

The difference in weight was 0.47 kg (95%CI −0.75, 1.70) in favor of the control group with high heterogeneity (I^2^ 87%) among the studies. The analysis was based on eight studies including 616 patients using gliclazide and 616 for the reference population [20], [23]–[27], [33], [36]. The treatment duration varied from 13 to 52 weeks.

When comparing gliclazide to other SUs or metiglinides the effect estimate was: −0.09 kg (95%CI −1.72, 1.55, I^2^ 87%). This analysis was based on four studies including 401 patients for gliclazide and 411 for SU or metiglinides [20], [27], [33], [36].

When comparing gliclazide to metformin the effect estimate was 1.37 kg (95%CI 0.15, 2.60, I^2^ 28%). This analysis was based on two studies including 45 patients for gliclazide and 48 for metformin [23], [25].

Studies with pioglitazone or DPP-4 inhibitors had a high risk for selective reporting; further weight comparisons with these individual drug classes were not performed.

### Cardiovascular events and mortality

The incidence of cardiovascular events could be evaluated in 9 studies [8], [23], [27], [28], [31]–[34], [36]. There were 11 cases in 1480 gliclazide users and 20 cases in the comparator group of 1508 patients, risk ratio for gliclazide 0.95 (95% CI: 0.57, 1.61). Information on total mortality and cardiovascular mortality was available from 17 and 15 studies, respectively. There were 12 deaths in 2500 gliclazide users and 8 deaths in the comparator group of 2569 patients, risk ratio gliclazide vs. others; 1.50 (95% CI: 0.62, 3.62) [8], [14], [20]–[29], [31]–[36]. The number of cardiovascular deaths were 3 in 1602 gliclazide users and 7 in 1619 comparator patients, risk ratio gliclazide 0.81 (95% CI: 0.26, 2.47) [8], [14], [20]–[28], [31]–[34], [36].

## Discussion

Severe hypoglycemic events caused by gliclazide are extremely rare and the occurrence of confirmed symptomatic non-severe hypoglycemic events in gliclazide users could exclusively be ascribed to studies using a gliclazide dose of 320 mg instead of the advised maximum dose of 240 mg. Gliclazide probably has advantages over glimepiride regarding hypoglycemia risk. Gliclazide appears to have a non-relevant beneficial effect on glycemic control compared to other oral glucose lowering agents. There were no trials investigating cardiovascular effects of gliclazide and weight comparisons were limited by a low number of trials and publication bias. The methodological quality of most studies was poor.

Gliclazide appears to be safe regarding severe hypoglycemia risk with one severe hypoglycemic event in 2,387 gliclazide users. Severe hypoglycemias were defined as the primary outcome, since severe episodes have the most deleterious effects [38]. We acknowledge that the risk of severe hypoglycemia in the comparator groups was also very low. This could either suggest that the risk of severe hypoglycemic events is rare in general, or that randomized trials are not the best way for evaluating hypoglycemic risk and that patients in trials represent a highly selected group.

The maximum dose of gliclazide (regular formulation) between studies varied from 240 to 320 mg. Two industry-sponsored high bias trials, both comparing gliclazide to vildagliptine, were responsible for all symptomatic confirmed non-severe events in gliclazide users; both used the 320 mg gliclazide dose [34],[35]. It appears that under the condition of not exceeding the 240 mg dose, gliclazide is safe regarding non-severe confirmed hypoglycemic events. One trial comparing gliclazide with glimepiride showed a definitely lower incidence of confirmed (either symptomatic or not) hypoglycemia events [29]. In patients who are at risk for hypoglycemic episodes, gliclazide is probably preferable to glimepiride.

Regarding hypoglycemic events, it should be noted that cut-off values can have great impact on the event rate [39] and non-symptomatic confirmed hypoglycemic events were not included in the meta-analyses because of concerns for overestimation of the event rate. The definition and recording of hypoglycemic events between studies differed substantially. Asymptomatic confirmed hypoglycemic events below for example 3.0 mmol/L could be relevant and we acknowledge that unconfirmed hypoglycemic events can be clinically relevant in patients with hypoglycemia unawareness.

There seemed to be a small benefit of metformin compared to gliclazide regarding weight although the effect estimate was small. Most studies either did not report or did not report ITT-analyses on differences in weight between individual drugs (i.e. pioglitazone and vildagliptine) and gliclazide, suggesting a high risk for publication bias in the weight analysis. The lack of cooperation by authors and pharmaceutical companies in providing data after our request is disconcerting [28], [30], [31], [34], [35]. Because of concerns for bias, weight comparisons between gliclazide and other individual drug classes, with for example DPP-4 inhibitors, were not performed.

Results from randomized trails in this review could not be used to confirm the observed beneficial cardiovascular effects found in cohort studies. The results regarding cardiovascular end-points and mortality must be interpreted with caution; none of the trials were designed to evaluate cardiovascular events, the number of randomized controlled trials included in the meta-analysis was low and the number of events was limited. Furthermore, study duration was relatively short in most cases. Also, a substantial number of studies did not report on specific adverse events. Randomized controlled trials are possibly not the best way of evaluating these adverse events, al least not when not designed for evaluating such endpoint and not endowed with a sufficiently long study duration [40].

A considerable amount of heterogeneity in the primary efficacy analysis was noted. This heterogeneity could be explained by differences in primary research questions [8], the inclusion of small studies, the use of different doses of gliclazide and varying use of diabetes drugs before randomization between studies.

We did not include the ADVANCE study in the meta-analyses. The ADVANCE investigated intense glucose control with gliclazide to a strategy with standard glucose control without gliclazide. Intensive glucose control itself (irrespective of the drug that is used) is associated with a lower HbA1c, increased risk for hypoglycemic events, weight increase and probably has beneficial effects on cardiovascular outcomes [37], [41].

Except for non-severe hypoglycemia risk, we were not able to make clinically relevant recommendations due a low number and quality of trails. Our conclusions regarding gliclazide as a second line treatment were not robust, due to a small number of trials.

We did not conduct separate analyses for each comparator class or looked at within-class difference because of scarcity of data. Furthermore, we did not conduct sensitivity analyses or meta-regression to examine the contribution of participants' baseline characteristics to the effect estimate of our primary outcome and did not conduct mixed treatment comparison/network meta-analysis. Exclusion of trials at high risk of bias in a sensitivity analysis did not relevantly alter the results of the main analysis, although this analysis included few trials.

## Conclusions

Relative few studies used gliclazide as active comparator despite years of clinical experience, no need for dose adjustment in renal dysfunction, low costs and observational studies showing possible cardiovascular benefits. The risk of severe or confirmed hypoglycemia was extremely low with gliclazide. Gliclazide could have a relative favorable short-term safety profile; specifically compared to glimepiride, under the condition of not exceeding the maximum dose of 240 mg, without evidence for a loss of efficacy. The quality of reporting changes in weight in randomized controlled trials could benefit from substantial improvements. Although none of the trials were designed to evaluation cardiovascular end-points, the possibility of cardiovascular benefits as shown in observational studies warrants attention in future randomized trials.

### Ethics Statement

An ethics statement was not required for this work.

### Data sharing

The full dataset and technical appendix are available at request from the corresponding author.

## Supporting Information

Attachment S1 **PROSPERO protocol.**
(TIFF)

Attachment S2 **Search strategy.**
(TIFF)

Attachment S3 **Risk of bias summary.** Presentation of the risk of bias summary of the review author's judgments about each risk of bias item for each included study.
(TIFF)

Attachment S4 **Risk of bias plot.** Presentation of the risk of bias graph of the review author's judgments about each risk of bias item presented as percentages across all included study. Studies in green or + are at low risk of bias.
(TIFF)

Checklist S1 **PRISMA Checklist.**
(PDF)
