# Supplementary figures and images for: Safety and Efficacy of Gliclazide as Treatment for Type 2 Diabetes: A Systematic Review and Meta-Analysis of Randomized Trials
Source: PLoS One. 2014 Feb 12;9(2):e82880. doi: 10.1371/journal.pone.0082880 (PMC3922704; doi:10.1371/journal.pone.0082880)

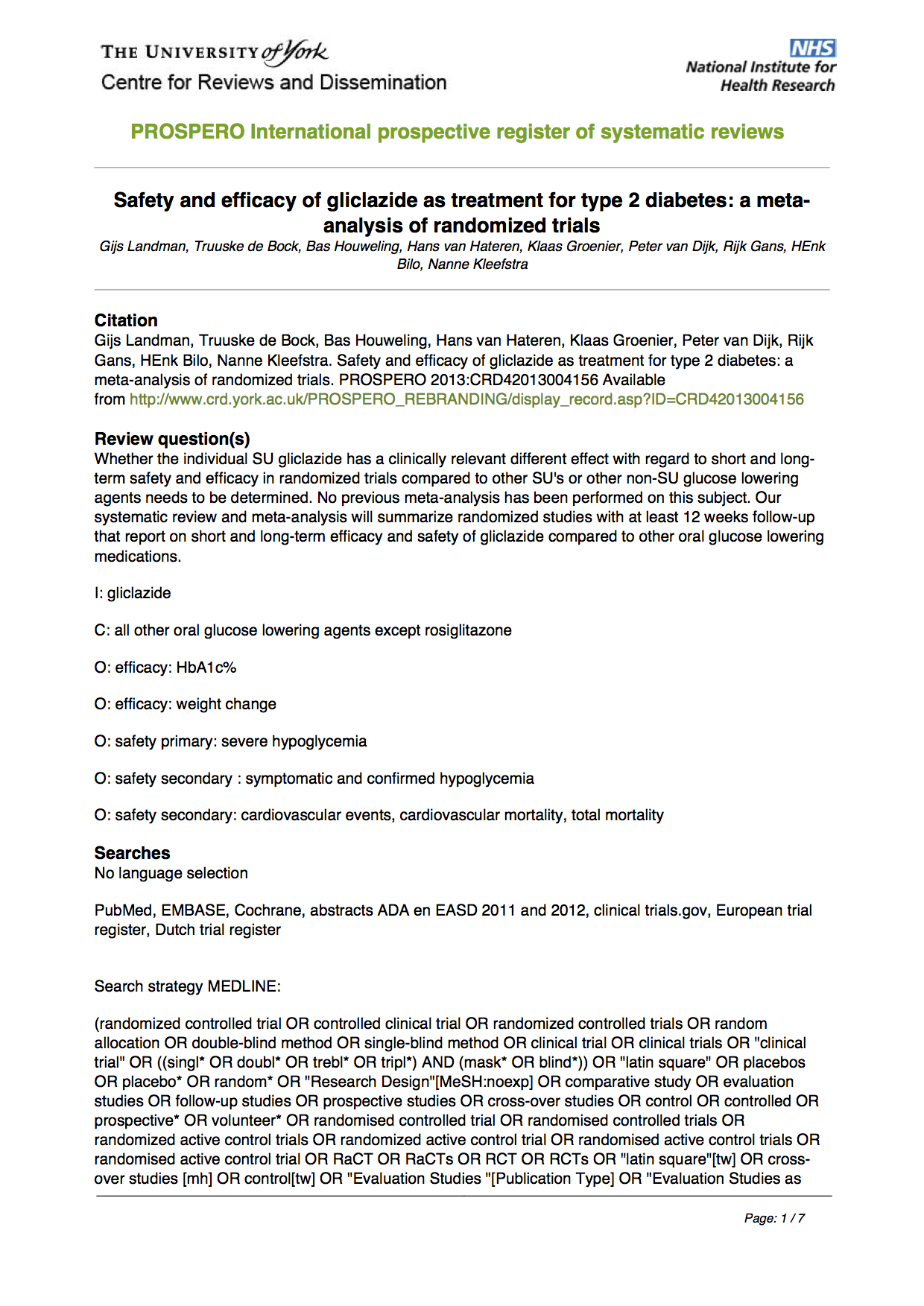

Supplement: Attachment S1 — PROSPERO protocol. (TIFF) [file pone.0082880.s001.tiff]

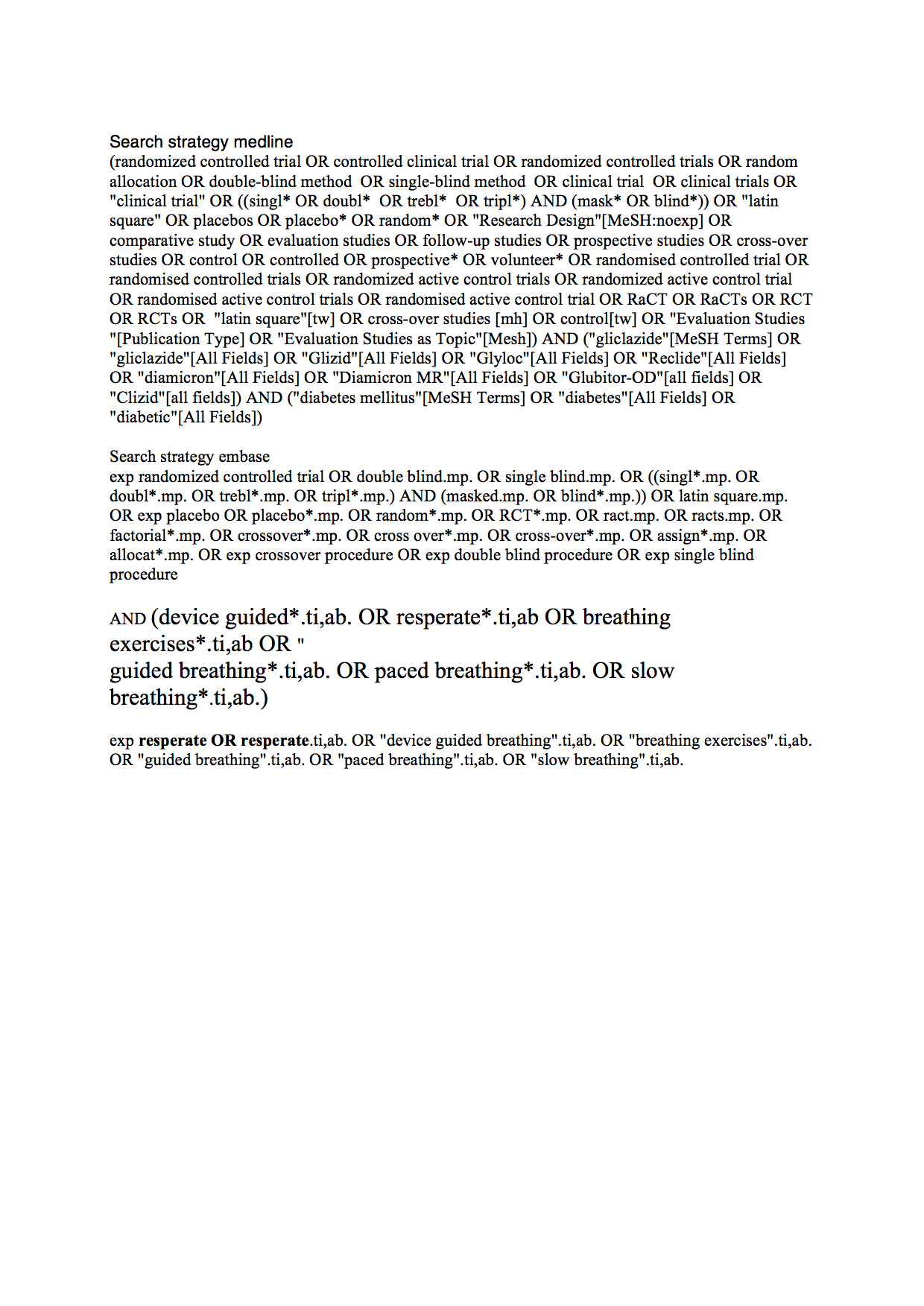

Supplement: Attachment S2 — Search strategy. (TIFF) [file pone.0082880.s002.tiff]

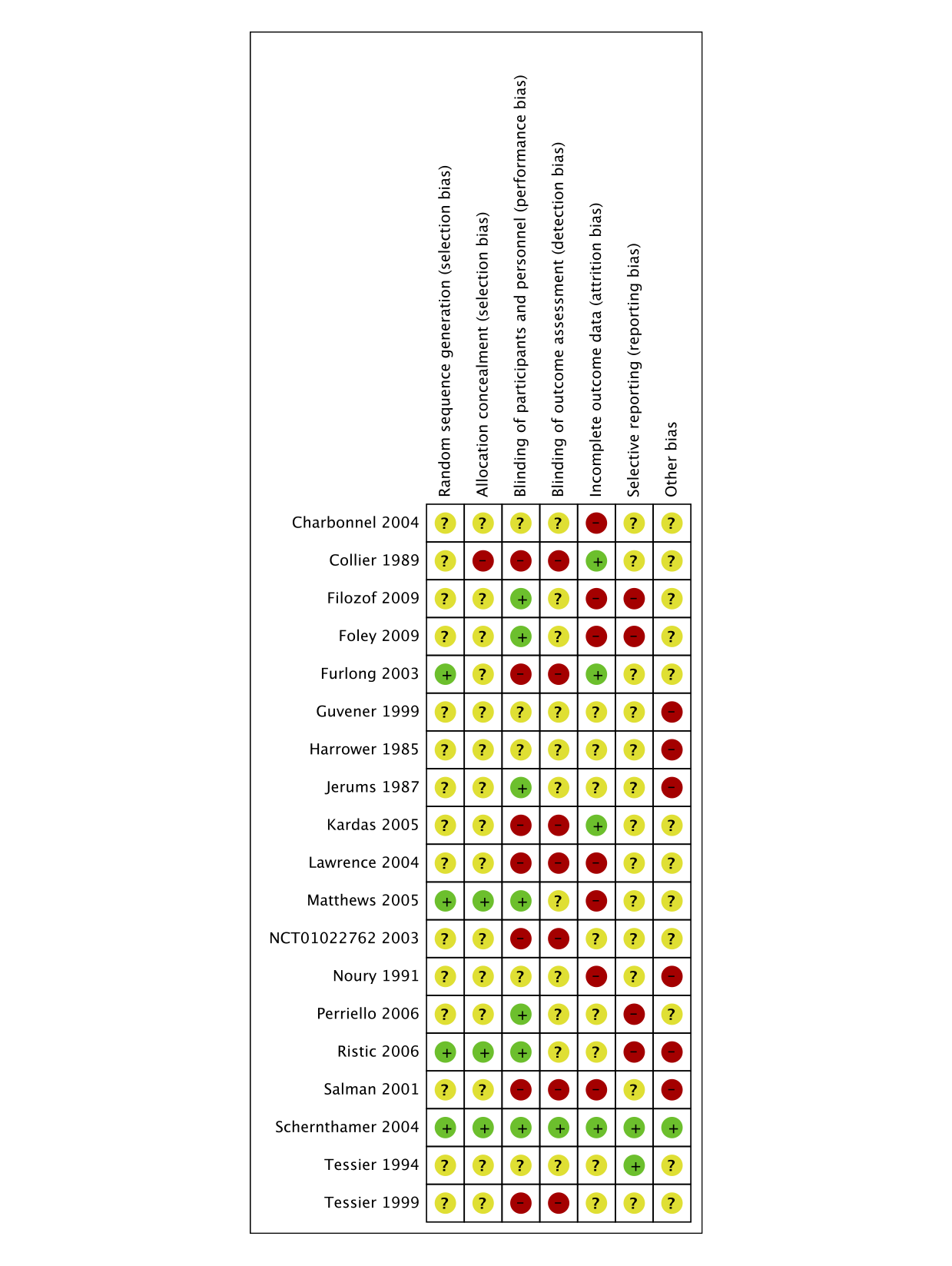

Supplement: Attachment S3 — Risk of bias summary. Presentation of the risk of bias summary of the review author's judgments about each risk of bias item for each included study. (TIFF) [file pone.0082880.s003.tiff]

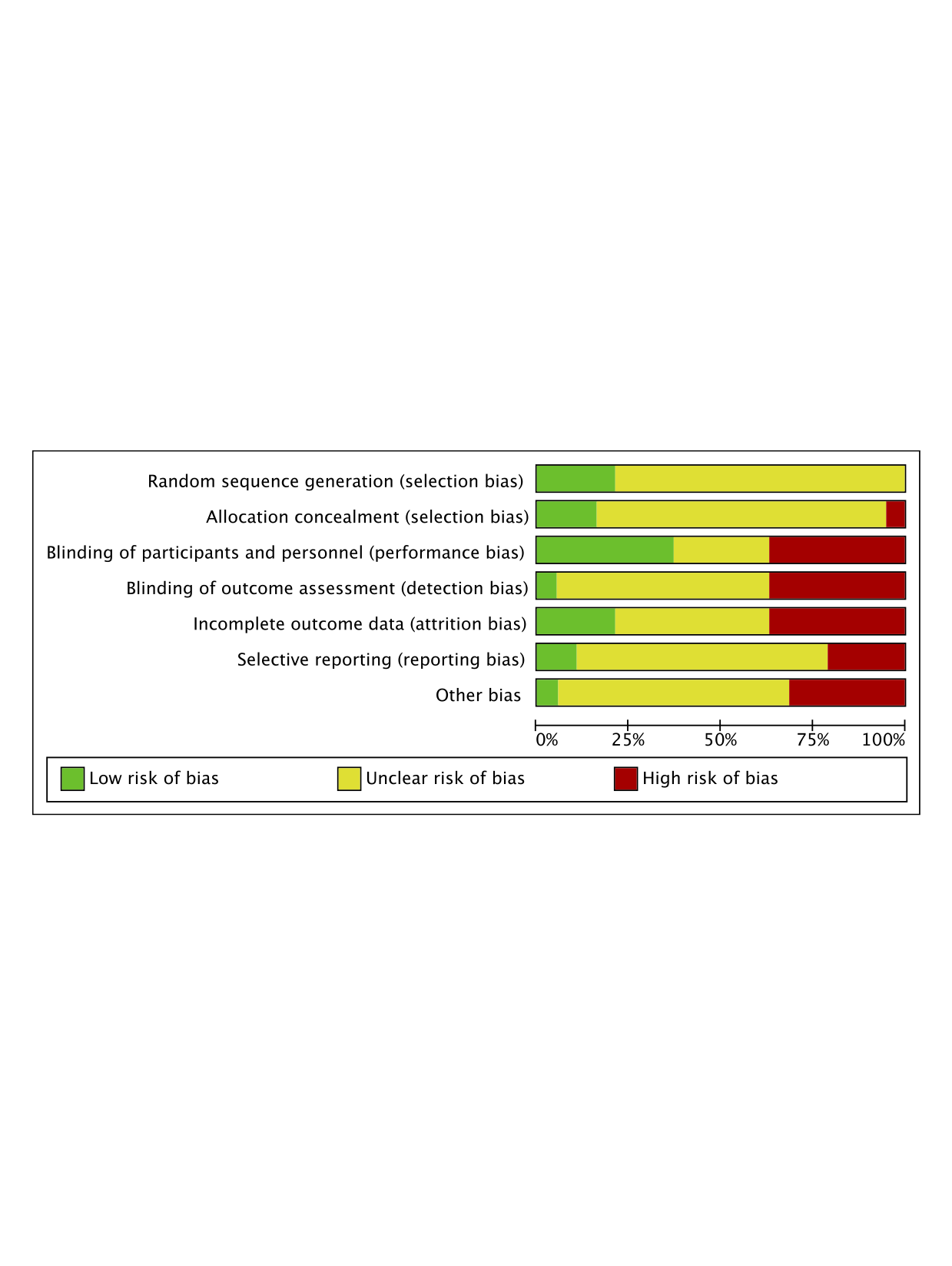

Supplement: Attachment S4 — Risk of bias plot. Presentation of the risk of bias graph of the review author's judgments about each risk of bias item presented as percentages across all included study. Studies in green or + are at low risk of bias. (TIFF) [file pone.0082880.s004.tiff]
